# Hydatidose cardiaque et rénale - apport de l'imagerie à propos d'une observation

**DOI:** 10.11604/pamj.2014.18.153.2410

**Published:** 2014-06-18

**Authors:** Abdennassar El Kharras, Mehdi Atmane, Jamal El Fenni, Souad Chaouir, Touria Amil

**Affiliations:** 1 Services d'Imagerie Médicale, Hôpital Militaire d'Instruction Mohamed V Rabat, Maroc

**Keywords:** Hydatidose, cœur, rein, imagerie, hydatidosis, heart, kidney, imagery

## Abstract

L’échinococcose cardiaque est rare, elle représente 0.2 à 2% des cas d'hydatidose. Elle siège essentiellement au niveau du ventricule gauche. La localisation péricardique est exceptionnelle. L’échocardiographie et la tomodensitométrie jouent un rôle primordial et sont généralement suffisantes pour poser le diagnostic de cette affection. Nous rapportons une observation d'une association de deux localisations peu courantes d'hydatidose rénale et péricardique partiellement rompu et l'intérêt de l'imagerie dans le diagnostic de ces lésions kystiques.

## Introduction

L'hydatidose péricardique est rare, même en pays d'endémie, de symptomatologie variée déroutant souvent le diagnostic. La présente observation illustre l'intérêt de l'imagerie en coupes dans le diagnostic et le bilan lésionnel d'une association exceptionnelle d'une hydatidose péricardique et rénale.

## Patient et observation

Mr HL âgé de 58 ans, tabagique est admis aux urgences pour des douleurs thoraciques rétrostérnale irradiant vers l’épaule gauche survenues lors d'un effort physique, accompagnées de nausées et de sueurs. L'examen clinique a retrouvé un patient apyrétique en bon état hémodynamique avec assourdissement des bruits du c'ur à l'auscultation cardiaque et une ischémie sous épicardique apico latérale sur l'ECG. La radiographie thoracique a montré un aspect légèrement globuleux de l'arc inférieur gauche. Le bilan biologique standard était sans particularité en dehors d'une hyperéosinophilie à 15%.

**Figure 1:**
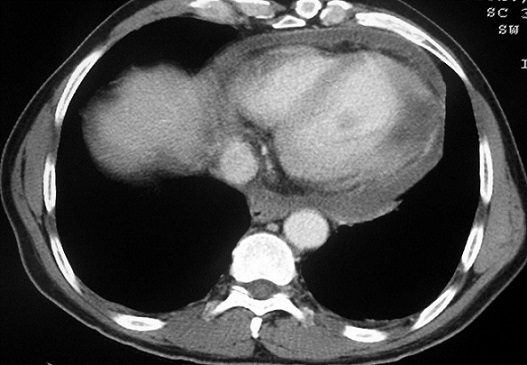
TDM thoracique en coupes axiales après injection intraveineuse d'iode: Masse kystique de la paroi latérale du ventricule gauche de contours mal définis non modifiée par le produit de contraste iodé associée à un épanchement péricardique

**Figure 2:**
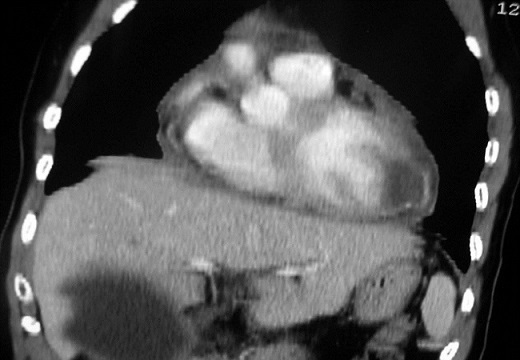
TDM avec reconstructions coronales: Paroi épaisse non rehaussée par le contraste siège de calcifications arciformes

**Figure 3:**
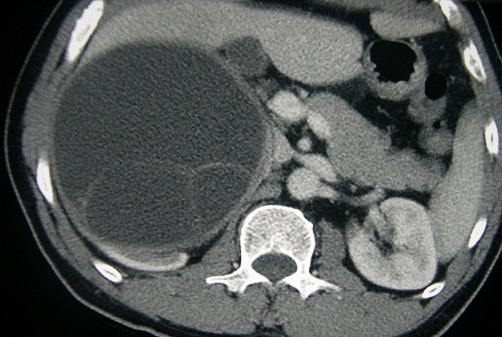
TDM abdominale en coupes axiales avec injection intraveineuse d'iode: Enorme masse kystique du pôle supérieur du rein droit à paroi épaisse renfermant des cloisons refoulant la face inférieure du foie

Le patient fut admis en cardiologie pour angor d'effort. L’échocardiographie trans thoracique a objectivé la présence d'un processus tumoral kystique multivésiculaire siégeant au niveau de la paroi latérale du ventricule gauche occupant toute l’épaisseur du segment moyen et apical avec trouble de la contractilité a ce niveau et un épanchement péricardique de moyenne abondance. Le scanner a mis en évidence une formation hypodense, liquidienne, homogène, grossièrement ovalaire mesurant 50×30mm environ, à paroi discrètement épaisse, non modifiée par le produit de contraste et calcifiée par endroit avec épanchement péricardique (Figure 1, Figure 2). Les coupes sous diaphragmatiques ont révélées la présence d'une énorme formation kystique rénale droite arrondie de 13.5 cm de diamètre, à paroi épaisse ne prenant pas le contraste et siège de fines cloisons (Figure 3). Ce processus refoule le pédicule rénal en arrière mais sans retentissement sur les voies excrétrices urinaires. Dans ses antécédents, le patient rapporte que son fils a été opéré il y a 6 mois pour une hydatidose hépato rénale.

Devant ce contexte clinique et radiologique le diagnostic de kystes hydatiques cardiaque et rénal grade III selon la classification de Gharbi (multivésiculaire) a été posé et ensuite confirmé par la chirurgie. Le patient a bénéficié d'une sternotomie sous CEC puis résection d'un kyste hydatique épicardo péricardique rompue partiellement dans le péricarde suivi d'un lavage au sérum salé hypertonique. Les suites opératoires ont été simples sans complications particulières. Actuellement, le patient est transféré en urologie pour la prise en charge du kyste hydatique rénal.

## Discussion

Même en zone d'endémie, la localisation cardiaque de l'hydatidose figure parmi les plus rares. L'incidence de cette localisation est faible de 0.5 à 2% [1] de toutes les localisations viscérales et provient essentiellement de la circulation coronarienne après franchissement du filtre hépatique et pulmonaire [2]. L'atteinte myocardique du ventricule gauche est la plus fréquente en raison de sa riche vascularisation pariétale et représente 55 à 60% des localisations cardiaques alors que celle du ventricule droit est de 15%, l'oreillette gauche 8%, l'oreillette droite 3 à 4%, le septum interventriculaire 7 à 9% [3]. La localisation péricardique comme dans notre cas est exceptionnelle [4] et semble toujours secondaire. Cette localisation est due soit à une rupture simple de l′adventice d′un kyste hydatique ventriculaire gauche avec chute de la vésicule intacte dans le sac péricardique: c′est l′échinococcose hétérotopique, soit à une rupture du kyste lui-même avec chute des vésicules filles: c′est l′échinococcose secondaire [5, 6].

La présentation clinique de l'hydatidose cardiaque est non spécifique et variée, elle dépend de la topographie, des dimensions, du nombre et de l'intégrité du kyste [7, 8]. La croissance des kystes hydatiques est souvent lente et asymptomatique, mais peut se manifester par des palpitations, une dyspnée d′effort, des précordialgies ou un angor [5, 9], comme c’était le cas chez notre patient qui a présenté un angor d'effort, ou une insuffisance valvulaire par compression ou envahissement. La rupture est rare mais souvent mortelle par choc anaphylactique, tamponnade, embolies massives cérébrales ou pulmonaires [10]. Sur le plan biologique, l′hyperéosinophilie et l′intradermoréaction de Casoni n'ont qu′une valeur d′orientation. L′immunofluorescence indirecte et l'ELISA sont les tests les plus sensibles alors que l′immunoélectrophorèse est le test le plus spécifique [11]. La sérologie hydatique n′est positive que dans environ la moitié des cas d’échinococcose cardiaque [5]. La radiographie thoracique détecte uniquement les formes volumineuses responsables d'une déformation de la silhouette cardiaque avec d’éventuelles calcifications pariétales curvilignes, arciformes ou en plaques et parfois une éventuelle localisation pulmonaire associée [12]. L’échocardiographie par voie trans-thoracique est l'examen de première intention du fait de son innocuité, de sa facilité d'utilisation et de son bon rendement diagnostique car elle permet de reconnaître le caractère liquidien et la paroi fine du kyste et d'en préciser la topographie [13]. L'image d'un décollement membranaire ou de vésicules filles, comme chez notre patient, est fortement évocatrice du diagnostic, mais rarement observée. Elle permet également de préciser l'existence d'un épanchement péricardique ou pleural associés. L’échographie abdominale doit être systématique à la recherche d'autres localisations hydatiques [5].

La tomodensitométrie, surtout multicoupes avec synchronisation cardiaque, permet un bilan morphologique et d'extension précis grâce à sa très bonne résolution spatiale et aux possibilités de reconstructions multiplanaires [10]. Le kyste hydatique se traduit généralement par une masse de densité liquidienne, bien limitée, arrondie, a paroi fine, non modifiée par le contraste. Elle est en général univésiculaire, exceptionnellement multivésiculaire. Les calcifications pariétales bien visualisées sont inconstantes mais évocatrices [5]. L'intérêt du scanner est la possibilité d'effectuer dans le même temps le bilan d'extension de la maladie par une acquisition thoraco-abdominale à la recherche de localisation multiviscérale (40% des cas), ce qui a été proposé dans notre observation par la découverte fortuite d'une localisation rénale [14].

L'IRM permet une caractérisation tissulaire de la masse et de mieux préciser la topographie et les rapports avec les organes de voisinages grâce aux coupes multiplans. Sur les séquences morphologiques pondérées en T1, le contenu du kyste est le plus souvent homogène hypo ou isointense au myocarde. La nature liquidienne est par contre d’évaluation plus difficile en raison des difficultés à obtenir une bonne pondération T2 sur les séquences cardiaques nécessitant une synchronisation électrique (ECG) et respiratoire, ce qui semble désormais possible grâce aux nouvelles séquences rapides de fast spin écho. Le retentissement fonctionnel du kyste sur la paroi touchée, sur les voies d’éjection et de remplissage et sur les structures en mouvement de voisinage est bien apprécié sur les séquences rapides en mode ciné et en apnée [10, 15].

## Conclusion

L'imagerie cardiaque en coupes apporte des informations intéressantes dans le bilan d'extension du kyste hydatique intracardiaque. Elle permet d'apprécier les possibilités de réparation chirurgicale et l’évolutivité des lésions après un diagnostic initial fait par échocardiographie.
